# Investigation of the efficacy of different Ni-Ti systems on acrylic blocks for correcting ledge formation

**DOI:** 10.1186/s12903-023-02865-8

**Published:** 2023-03-25

**Authors:** Osman Ünlü, Hüseyin Gürkan Güneç, Faruk Haznedaroğlu

**Affiliations:** 1 Faculty of Dentistry, Department of Endodontics, Istanbul University, Prof. Dr. Cavit Orhan Tütengil Sk. 4/6, 34116 İstanbul, Turkey; 2 Hamidiye Dental Faculty, Department of Endodontics, University of Health Sciences, Tıbbiye Cd, 34668 Selimiye, Üsküdar, İstanbul, Turkey

**Keywords:** Ledge, Procedural error, Stereomicroscope, Fanta AF™ LC, HyFlex EDM

## Abstract

**Background:**

The aim of this study was to compare the efficacy of K-type stainless steel hand instruments (Mani Inc. ), Fanta AF™ Ledge Correction (LC) (Fanta Dental), and Hyflex EDM (Coltene-Whaledent) for ledge correction, canal transport, centric ability, and shaping (preparation) time after an artificial ledge has been bypassed manually in highly curved canals using acrylic blocks.

**Methods:**

Forty-two resin blocks, each with a radius of 5 mm (Endo Trainer Block, VDW) and an apical inclination of 55°, were used. Under stereomicroscope magnification, standard artificial ledges were created on acrylic blocks, and attempts were then made to eliminate them using hand instruments, FantaAF™ LC, and Hyflex EDM. Before and after images were obtained using a stereomicroscope and compared using Photoshop.

**Results:**

Fanta AF™ LC and Hyflex EDM were found to be more effective for correcting ledges than hand instruments. The use of hand instruments resulted in the greatest transportation away from the canal curvature in the apical area. The canal shaping was completed in the shortest amount of time using Fanta AF™ LC, followed by HyFlex EDM and then the hand instruments.

**Conclusion:**

In terms of centric ability, the order from best to worst is as follows: Fanta AF™ LC, Hyflex EDM, and hand instruments. After the ledge was manually bypassed with hand instruments in the root canals, Hyflex EDM and Fanta AF™ LC were found to be more effective than hand instruments in reshaping the previously unreachable region between the ledge and the foramen apical.

## Background

The success of endodontic treatment depends on effective biomechanical preparation and subsequent hermetic root canal filling. Improper use of instruments during root canal treatment, use of metallurgically defective instruments, and difficult canal anatomy are factors that lead to procedural errors, such as zip and ledge perforation [1, 2]. Ledge formation is especially prevalent in narrow and curved root canals. Kapalas and Lambrianidis showed that 52% of canal treatments administered by students had ledge formation [3]. In root canal treatments performed by endodontists, ledges occurred in 33% of teeth that received initial root canal treatment and in 41% of teeth that received retreatment. According to Eleftheriadis and Lambrianidis, 25% of root canals performed by dentistry students had ledges [4]. The possible causes of ledge formation include an inadequate access cavity, failure to provide straight-line access, incorrect determination of working length and root canal alignment, forcing the instrument into the canal, using a noncurved stainless steel instrument that is too large for a curved canal, failing to use the instruments in sequential order, and inadequate irrigation during shaping [3, 5].

Ledge formation might adversely affect the outcome of endodontic treatment. Especially in infected teeth, the inaccessible necrotic pulp located apically to the ledge can result in persistent apical periodontitis. To ensure a successful treatment outcome in such cases, the working length should be re-established, and effective biomechanical preparation should be applied to the apical foramen after the ledge has been bypassed. Thin K-files with a precurvature of 45° are used with copious irrigation to bypass ledges. Reshaping can be performed using hand instruments or Ni-Ti instruments once the ledge has been bypassed. The aim is to eliminate the ledge as much as possible to provide adequate irrigation and a bacteria-tight root canal filling [6, 7].

Several new-generation NiTi files were designed and marketed for endodontic treatments using a single-file technique in perpetual rotations. The HyFlex EDM is one of the cutting-edge instruments produced for this purpose, which is manufactured using the technique of electrical discharge machining with a controlled-memory feature [8–10]. Another important feature of the Hyflex EDM system is the file design with different cross-sections along the cutting surface. The coronal region is triangular to provide better cutting properties. Its middle region is trapezoidal, which provides better resistance and better removal of dentin residues, while its tip is rectangular, which provides better grip and reduces the risk of fracture [9].

A Ni-Ti called Fanta AF™ Ledge Correction (LC), which has a special tip that has been designed specifically for use in ledge-containing canals, has recently been introduced. Control memory Ni-Ti instruments have demonstrated superior results compared to conventional Ni-Ti instruments in terms of effective cleaning in curved and narrow canals and maintaining the original shape of the canal [11].

This study aims to compare the efficacy of hand instruments, Fanta AF™ LC, and Hyflex EDM for ledge correction, canal transport, centric ability, and application time in highly curved acrylic blocks with artificial ledges after the ledge has bypassed manually. Our null hypothesis is that there is no difference between these methods.

## Methods

### Sample preparation

The study was carried out on acrylic blocks on which artificial steps were created. In order to determine the required number of samples, the effect size for Change in angle of canal curvature (degree) was found to be 0.50 among the study groups in the power analysis performed with the G*power 3.1 program (alpha error probability = 0.05); In the sample size analysis performed by taking the power value of 0.80, the total number of samples required to be taken in total was found to be 42 (n:14 for each group).

Forty-two resin blocks, each with a radius of 5 mm and an apical curvature of 55°, were used (Endo Trainer Block, VDW, Munich, Germany). After coronal flaring, size 35, 40, and 45 SS K-files were advanced into the canal with pressure and ledge was created at the beginning of the curvature. The ledge prepared by a single operator with an K file was fixed at 13.5 mm of the canal with the help of a stopper, and its width was examined under stereomicroscope magnification (x20) with a width of 450 µ (+-10 µ).

All operations were conducted by a single operator. The blocks were covered with dark tape so that the operator could not see which sample was being worked on or the direction and position of the ledge. The acrylic blocks were numbered and randomly divided into three groups, one control and two experimental groups, each containing 14 samples.

In all groups, a stainless steel #10 file type K (Mani Inc., Tochigi, Japan) with a precurvature of 45° was used to bypass the ledge with a slight in-and-out pecking motion while the canal was filled with an irrigation solution. Then, a #15 type K file was used to create a glide path. In our study, files were used just once. After each file, the canal patency was checked using a #10 type K file and irrigated using 2 mL of distilled water administered with a 30-gauge (G) perforated irrigation needle. The final irrigation of the canal was completed using 5 mL of distilled water.

### Experimental groups

#### Control group

In this group, after bypassing the ledge, precurved #20, #25, and #30 K files were used, and the MAF was determined to be #30. Then, using anti-curvature filing with the step-back method, size #35, #40, and #45 K files that were one mm shorter than the working length were used. The following are descriptions of the procedures carried out for each group.

#### Group 1

In this group, the ledges were bypassed, and glidepaths were established until size #15 was reached, as in the control group. Shaping was performed using Fanta AF™ LC (Fanta Dental, Shanghai, China). After the rigid precurved tip bypassed the ledge, it was operated using an endo motor (Fanta Dental, Shanghai, China) following the manufacturer’s suggestions. Fanta AF™ LC files 10/06, 15/06, 20/07, and 25/08 were used with 3 N/cm of torque and a reciprocal motion of 350 rpm (90° clockwise and 30° counter clockwise). Push-pull movements of 1–2 mm were made until the working length was reached. Meanwhile, attempts were made to eliminate the ledge by applying pressure.

#### Group 2

In this group, as in the control group, the ledges were bypassed, and glidepaths of #15 were established. The HyFlex EDM (Coltene-Whaledent, Altstätten, Switzerland) file was precurved, and after the ledge had been bypassed, it was operated using an endo motor. Then, the HyFlex EDM was rotated at 500 rpm with 2.5 N/cm of torque, was used with 10/05, 20/05, and 25 ~ files. A push-pull motion of 1–2 mm was employed until the working length was achieved while pressure was applied to the ledge in an attempt to eliminate it.

The quality of the shaping performed and the evaluation of whether the ledge was corrected were determined based on whether there was improvement or no improvement. The absence of the ledge’s edge and the continuation of the canal in a straight line were interpreted as an improvement. There was considered to be no improvement if the edge of the ledge was visible after shaping. In addition, canal transportation after the final shaping was evaluated. After shaping, the changes were measured in acrylic blocks with artificial ledges based on the following criteria [12, 13].

### Measurement criteria

***Canal transportation***: Images were compared before and after shaping, and the difference was calculated. Measurement of the amount of transportation by A was measured as follows: (a1 - a2). The amount of transportation by B was measured as follows: (b1 - b2).

**Fig. 1:**
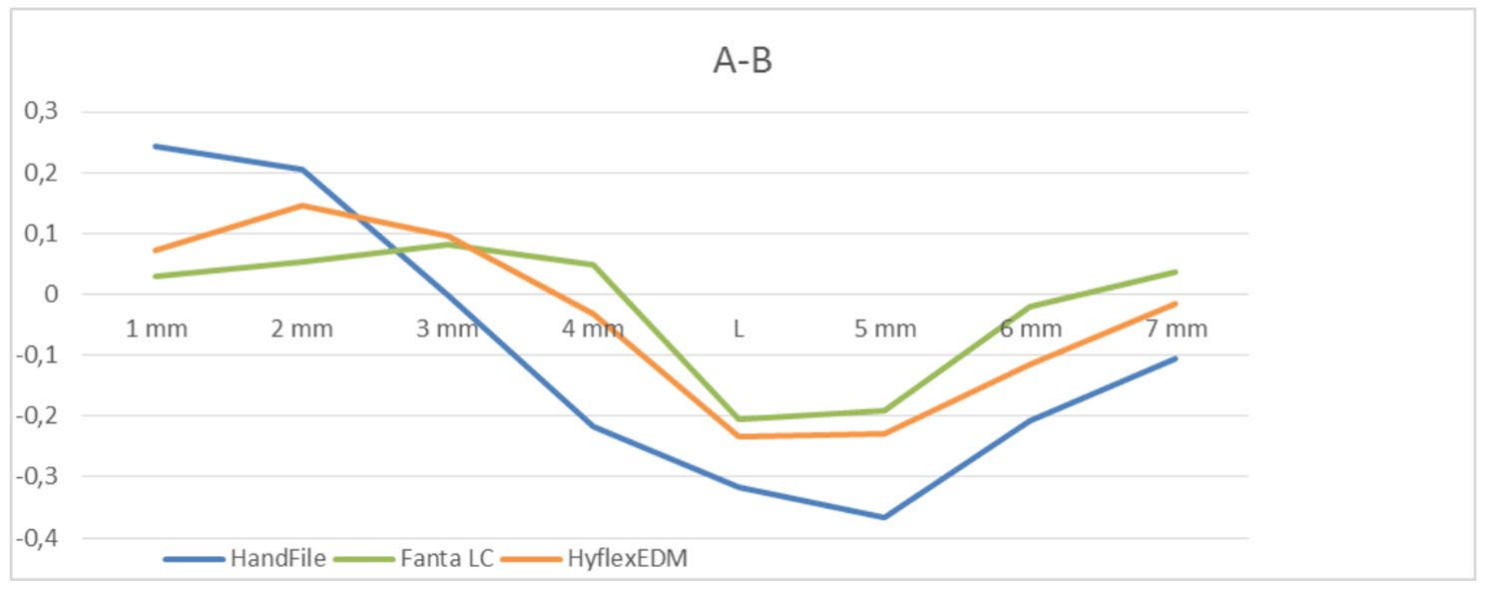
Direction of transportation

***Direction of transportation***: This was identified as the point at which more material was removed from the curvature. (Amount of substance removed by B - Amount of substance removed by A) (Fig. 1).

***Centric ability***: This was calculated by dividing the amount of material removed from the smaller side of the canal by that removed from the larger side. [(a1 - a2)/(b1 - b2) or (b1 - b2)/(a1 - a2)] If these numbers were not equal, the smaller one was considered a fraction of the ratio. A result of 1 indicates a perfect centring ratio [14].

***Operation time***: After bypassing the standard ledge in all groups and reaching the working length, the amount of time required to complete apical shaping was measured using a stopwatch. The measured time period included all the operations, such as the insertion of the instruments in the canal, the removal and cleaning of the threads, changing the instruments, and irrigation, until the completion of apical shaping.

To standardize the ledges created in acrylic blocks and to capture images before and after shaping, an Olympus SZX7 (Japan) stereomicroscope was used.

**Fig. 2:**
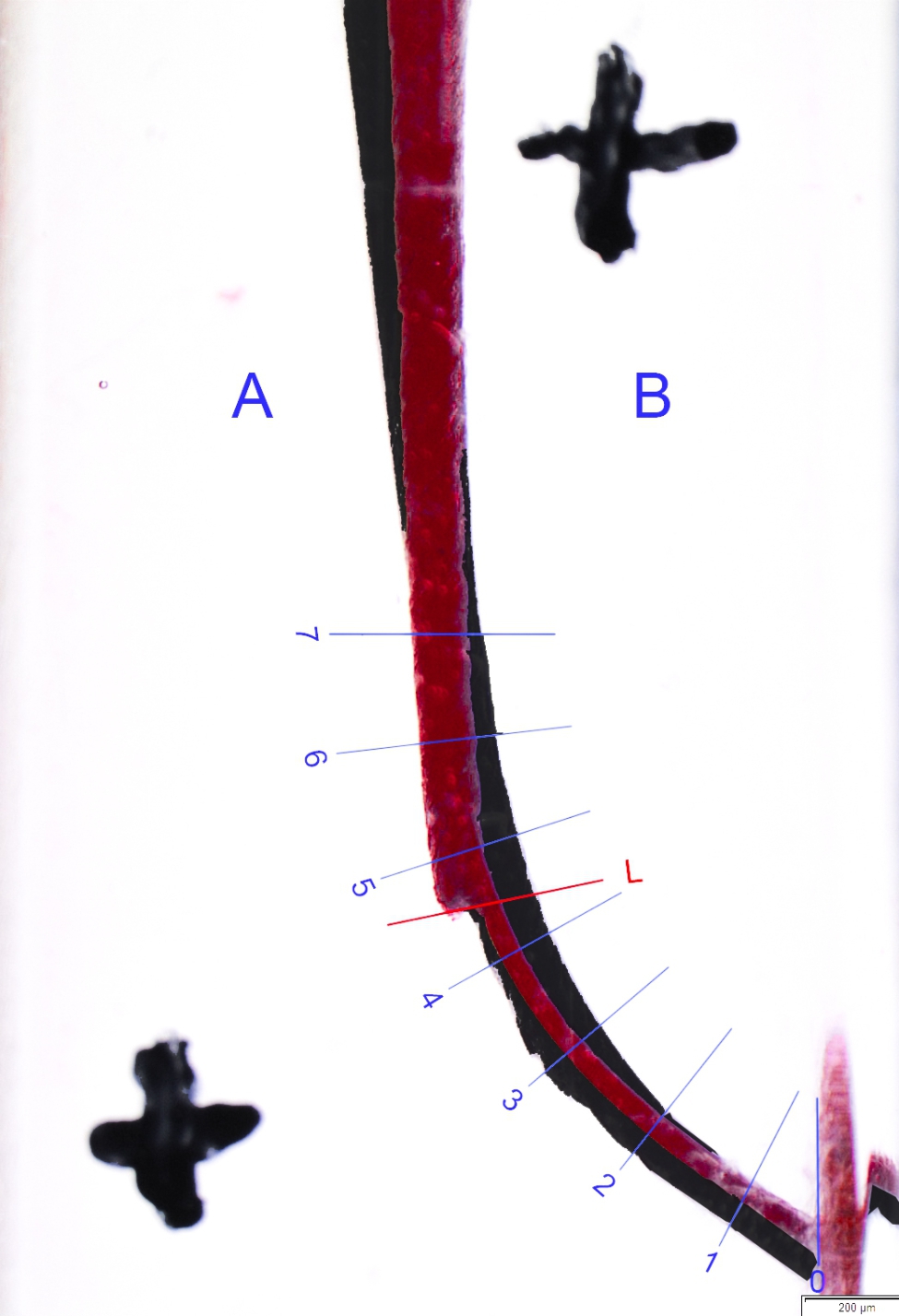
Superimposition of before preparation and after preparation Hand File’s images on endotraining blocks by using Adobe Photoshop

**Fig. 3:**
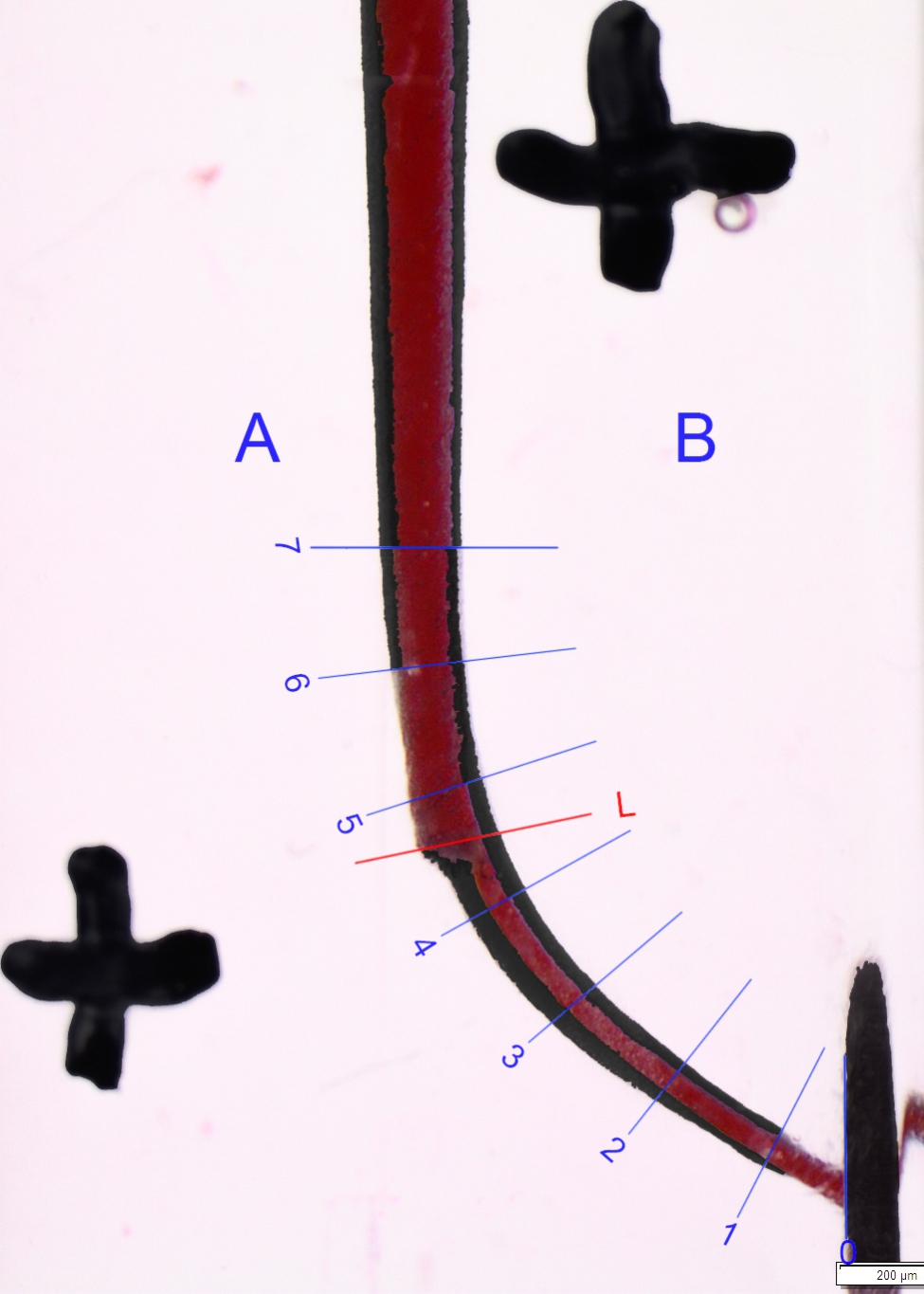
Superimposition of before preparation and after preparation Fanta AF™ CL’s images on endotraining blocks by using Adobe Photoshop

**Fig. 4:**
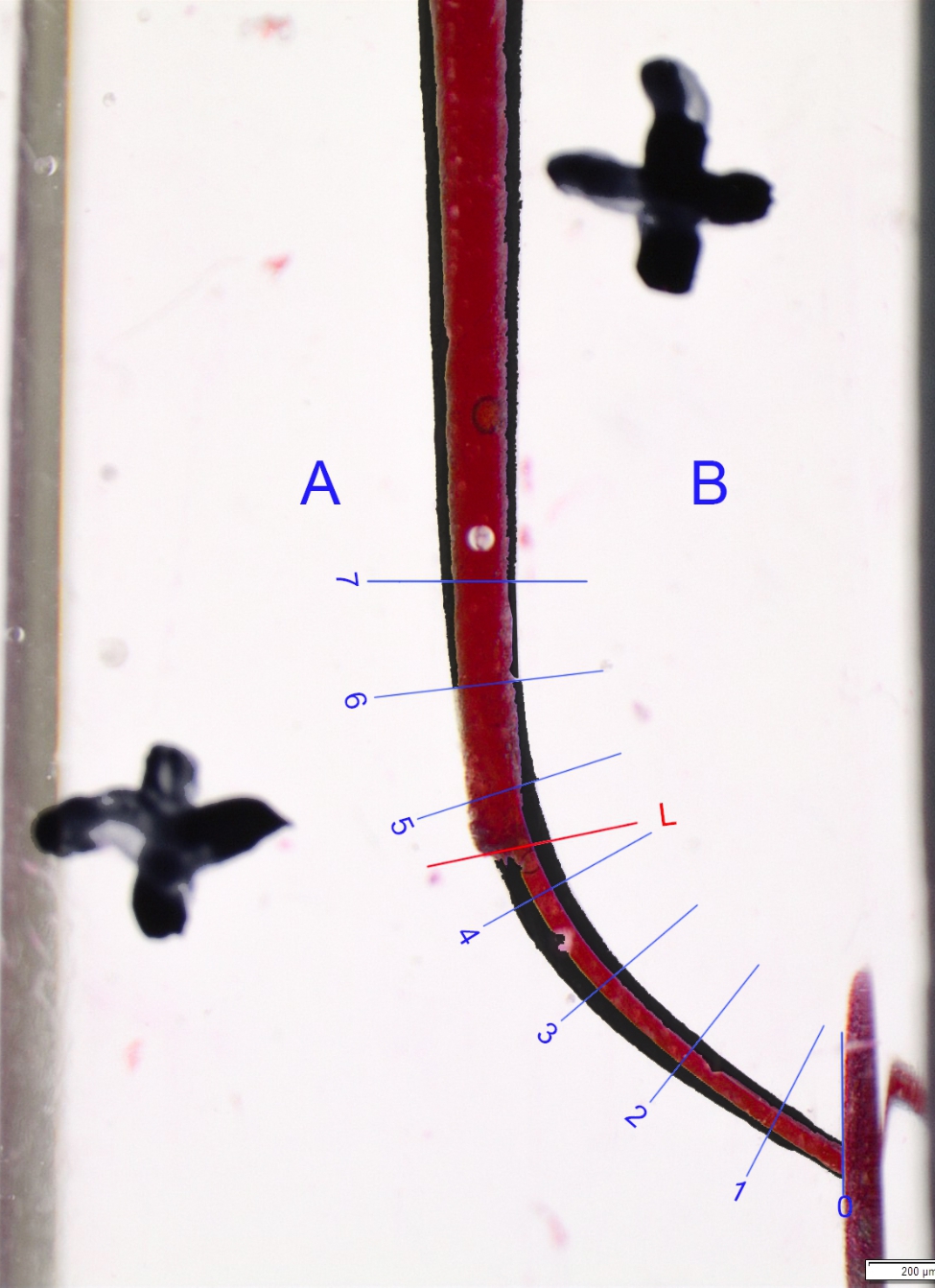
Superimposition of before preparation and after preparation Hyflex EDM’s images on endotraining blocks by using Adobe Photoshop

Images were captured using a stereomicroscope and a standard setup. The standardization of the photographs was ensured by applying two crosses. The blocks were painted with red ink before shaping and black ink after shaping to produce clearer results when the images were assessed using a computer program. Relevant data were overlapped with pixel sensitivity and compared using Photoshop (Adobe Inc./USA) software (Figs. 2, 3 and 4).

### Statistical analysis

SPSS version 17.0 was used to conduct statistical analyses. Using histogram graphics and the Kolmogorov-Smirnov test, the compliance of the variables with a normal distribution was investigated. Means and standard deviations were used for descriptive analysis. The Pearson chi-square test was used to compare categorical variables, the Kruskal-Wallis test was used to evaluate between-group variables that did not have a normal distribution (nonparametric), and the Mann-Whitney U test was used to make paired comparisons for significant results. The results with a p-value of less than 0.05 were considered statistically significant.

## Results

Our research qualitatively investigated whether a ledge could be corrected. While there was no improvement to ledges in any of the control group samples in which hand instruments were used, a 42.86% improvement was observed in experimental Group 1 (Fanta AF™ LC), and a 35.71% improvement was observed in experimental Group 2 (HyFlex EDM). Both experimental groups showed significantly superior improvement (p < 0.05) compared to the control group (hand instruments).

The control group’s average procedure time (7.936 ± 0.310 min) was statistically longer than Group 1’s (4.550 ± 0.340) and Group 2’s (5.502 ± 0.333) average times (p < 0.05). The procedure was completed more quickly in Group 1 than in Group 2, and the difference was statistically significant (p < 0.05).

When evaluating the material removal at the outer aspect of the curve (A) in the apical area, statistically higher values were observed in the control group (hand instruments) than in Group 1 and Group 2. The amount of material extracted from the outer canal curvature in the coronal region was lower in the control group than in Group 1 and Group 2, and the differences were statistically significant (p < 0.05) (Table 1).

**Table 1:** Average values of transportation of instrumentation groups in each section of artificial canals

		Outer (A)		p	İnner (B)		p
		Av.	s.s.		Av.	s.s.	
Apical	Hand File	0,254†	± 0,057	**0,001**	0,030	± 0,017	**0,001**
	Fanta AF™ CL	0,124	± 0,025		0,082†	± 0,026	
	Hyflex EDM	0,141	± 0,037		0,032	± 0,038	
Middle	Hand File	0,100†	± 0,038	**0,001**	0,295†	± 0,032	**0,001**
	Fanta AF™ CL	0,144	± 0,012		0,164	± 0,016	
	Hyflex EDM	0,131	± 0,030		0,186	± 0,047	
Coronal	Hand File	0,002†	± 0,006	**0,001**	0,158	± 0,035	**0,001**
	Fanta AF™ CL	0,092	± 0,033		0,085†	± 0,027	
	Hyflex EDM	0,064	± 0,033		0,129	± 0,047	

* p < 0.05

† Kruskal Wallis Test

When the amount of material extracted from the inner side (B) of the canal curvature was assessed, it was determined that Group 1 removed significantly more material in the apical region than the control group (hand instruments) or Group 2. The amount of material extracted from the middle region was greater in the control group (hand instruments) than in Group 1 and Group 2. The differences between the groups were statistically significant (p < 0.05) (Table 1).

When the rate of centric ability is examined; Group 1 (Fanta AF™ LC) at 1 and 2 mm levels; It is higher than the control group (Hand file) and Group 2 (Hyflex EDM). At 4 mm, 6 mm, 7 mm levels, the centeric ability rate is lower in the control group (Hand file), Group 1 (FantaAF™ LC) and Group 2 (Hyflex EDM) groups. The differences between the groups were statistically significant (p < 0.05). There is no statistically significant result at 3 mm, 5 mm and L levels. (p > 0.05). The centric ability was lower (0,172 ± 0,266) for the control Group than it was for Group 1 and Group 2. Fanta AF™ LC was determined to have the best centric ability (0,503 ± 0,365), followed by Hyflex EDM (0,347 ± 0,374) and then hand instruments. The differences found between the groups were statistically significant (p < 0.05) (Table 2).

**Table 2:** Centric ability values

Levels	Hand Files		Fanta AF™ LC		Hyflex EDM		P*
	Av.	±s.s.	Av.	±s.s.	Av.	±s.s.	
1 mm	0,006	± 0,024	0,660	± 0,348	0,161	± 0,279	0,001
2 mm	0,241	± 0,149	0,689	± 0,221	0,259	± 0,305	0,001
3 mm	0,722	± 0,170	0,614	± 0,165	0,574	± 0,287	0,239
4 mm	0,340	± 0,183	0,785	± 0,136	0,800	± 0,238	0,001
Ledge	0,043	± 0,116	0,000	± 0,000	0,023	± 0,087	0,350
5 mm	0,000	± 0,000	0,000	± 0,000	0,007	± 0,024	0,368
6 mm	0,000	± 0,000	0,660	± 0,369	0,199	± 0,206	0,001
7 mm	0,025	± 0,095	0,619	± 0,193	0,755	± 0,265	0,001
Average	0,172	± 0,266	0,503	± 0,365	0,347	± 0,374	0,001

* p < 0.05

## Discussion

To date, the utilization of precurved hand instruments has been considered the most effective approach to bypassing a ledge [5]. A considerable amount of time is generally spent distinguishing the original canal from the ledge, which is followed by the complete elimination of the ledge so that the canal can be properly obturated. This research compared the performances of the conventional method with the first file that has been manufactured to resolve this clinical problem, Fanta AF™ LC, a precurved reciprocal file, and HyFlex EDM, a controlled memory Ni-Ti system.

Acrylic blocks were selected for this study because they allow for simple, transparent observation of changes in the canal before and after shaping and standardizing the canal diameter, size, and curvature in three dimensions [15, 16]. It was performed on acrylic blocks because it would be difficult to create and observe ledges on extracted teeth. However, since all the experiments were performed under identical conditions, this is not considered to be a significant variable. According to Burroughs et al. and Pacheo-Yanes et al., who conducted similar studies, distilled water is the preferred irrigation solution due to the possibility that sodium hypochlorite might alter the structure of the acrylic block [17, 18].

Bergmans et al. and Gergi et al. compared stainless steel hand instruments with Ni-Ti instruments and discovered that when the apical shaping size was greater than #30, the amount of canal transportation increased [14, 19]. In our study, the extent of apical shaping was determined to be 30/02 for K files, 25/08 for Fanta AF™ LC files, and 25̴ for Hyflex EDM files, which was used to standardize the systems. If larger diameters were desired, standardization between instruments could not be achieved. In the Fanta AF™ LC system, the apical diameter of the larger file is #30, but its taper is 09. There is no file with an apical diameter of #30 in the Hyflex EDM system.

We were unable to identify a study in the literature, particularly a study conducted with canals with ledges, that was similar to ours. In addition, there have been no studies involving the Fanta AF™ LC file. Thus, our results may be compared to those of studies examining the efficacy of endodontic instruments in root canals in terms of duration, adherence to the canal’s original shape, and centric ability. Our results indicate that Groups 1 and 2 were more successful in terms of ledge elimination than the control group, and the differences were statistically significant. This may be explained by the high elasticity of Group 1 and Group 2 and the higher tapers (25/08, 25/ ~) compared to hand instruments.

In terms of working time, Fanta AF™ LC had the best results. Although Hyflex EDM was slower than Fanta AF™ LC, its working time was less than hand instruments by a statistically significant amount (p < 0.05). This finding is consistent with previous research indicating that reciprocal systems shape canals more quickly than rotational systems or hand instruments [20–23].

Çelik et al. compared six different Ni-Ti instruments with hand instruments and reported that hand instruments produced more canal transportation than all Ni-Ti systems [24]. Similar results have been obtained by many researchers [25–27]. In the control group, where hand instruments were used, the amount of canal transportation on the outer aspect of the curvature at the apical region was statistically significant compared to the experimental groups. There was more canal transportation at the concave part of the curvature in the area between 4 and 5, where the curve begins, and L, and the difference was statistically significant. Therefore, hourglass formation occurred at the apical region. This can be interpreted as resulting from the insufficiency of hand instruments in ledge correction, in that they remove excessive amounts of material from the inner side of the curvature compared to Ni-Ti instruments, which causes the canal to flatten. This may be due to the use of a rigid #30 K file, which is less flexible than Ni-Ti Fanta AF™ LC and Hyflex EDM, and the canal being severely curved. In terms of canal transportation in the outer aspect of the curvature, there was no statistically significant difference between Fanta AF™ LC and Hyflex EDM. In Group 1, the removal of more material from the inner side of the curvature at the apical region compared to the other two groups (the control group and Group 2) may be attributed to the precurved rigid tip of the instrument, which cleans the concave aspect of the curvature more effectively. The reason is that Hyflex EDM and Fanta LC files are heat treated and their cross-sections are triangular, while K file files are not heat-treated and have square cross-sections.

In terms of transportation, Hasheminia et al. reported that using an EdgeFile (EdgeEndo, Albuquerque, NM) operating with rotational motion caused less transportation than the other two systems (Reciproc, Wave One) operating with reciprocal motion [28]. In our study, there was no statistically significant difference in the total transportation between Hyflex EDM, a rotational file, and Fanta AF™ LC, a reciprocal file.

Kumar et al. reported that hand instruments displayed less centric ability than the other two Ni-Ti rotary instrument systems (Twisted File and Hyflex Cm) that they compared [29]. According to them, this may be due to the rigidity of stainless steel hand instruments and their strong cutting ability. This finding is consistent with the results of our study. In line with the current literature, both Ni-Ti systems were found to be more successful in terms of centric ability than stainless steel hand instruments. Between the two Ni-Ti systems, Fanta LC was found to be superior to HyFlex EDM, probably due to its precurved rigid tip and flexibility.

## Conclusion

Fanta AF™ LC completed canal shaping in the shortest amount of time, followed by the HyFlex EDM group and then the hand instruments control group. The hand instrument group had the longest working time. When the three groups were evaluated in terms of canal transportation occurring in the apical 1/3 after shaping, the most transportation was seen in the hand instrument control group. Although Fanta AF™ LC induced more transportation on the inner side of the curvature than HyFlex EDM, the transportation was similar between the two Ni-Ti systems. The most successful results in terms of centric ability were obtained using Fanta AF™ LC, followed by HyFLex EDM and then hand instruments.

## Data Availability

The datasets used and/or analyzed during the current study are available from the corresponding author on reasonable request.

## Declarations

### Clinical relevance

One of the complications that can be experienced in endodontic practice is the formation of ledges. In this difficult process, which directly affects the success of the treatment, practitioners sometimes cannot correct this ledge, and therefore, the tooth whose treatment cannot be completed can be extracted.

## References

[1] Alamoudi RA, Alharbi AH, Farie GA, Fahim O (2020). The value of assessing case difficulty and its effect on endodontic iatrogenic errors: a retrospective cross-sectional study. Libyan J Med.

[2] Bhuva B, Ikram O (2020). Complications in endodontics. Prim Dent J.

[3] Kapalas A, Lambrianidis T (2000). Factors associated with root canal ledging during instrumentation. Endod Dent Traumatol.

[4] Eleftheriadis GI, Lambrianidis TP (2005). Technical quality of root canal treatment and detection of iatrogenic errors in an undergraduate dental clinic. Int Endod J.

[5] Jafarzadeh H, Abbott PV (2007). Ledge formation: review of a great challenge in endodontics. J Endod.

[6] Gabardo MCL, da Silva WJ, Gonçalves LM, Deonízio MDA (2013). Effectiveness of different obturation techniques in surpassing the ledge formed in simulated curved canals. Braz J Oral Sci.

[7] Adina S, Irina-Maria G, Loredana M, Mitran M, Anca-Nicoleta T, Liviu G, Ruxandra S, Paula P (2018). Assessment of three techniques in surpassing Ledges in Curved Canals. ARS Med Tomitana.

[8] Yilmaz K, Uslu G, Ozyurek T (2017). In vitro comparison of the cyclic fatigue resistance of HyFlex EDM, one G, and ProGlider nickel titanium glide path instruments in single and double curvature canals. Restor Dent Endod.

[9] Pirani C, Iacono F, Generali L, Sassatelli P, Nucci C, Lusvarghi L (2016). HyFlex EDM: superficial features, metallurgical analysis and fatigue resistance of innovative electro discharge machined NiTi rotary instruments. Int Endod J.

[10] Bhatt A, Rajkumar B (2019). A comparative evaluation of cyclic fatigue resistance for different endodontic NiTi rotary files: an in-vitro study. J Oral Biol Craniofac Res.

[11] Shen Y, Zhou HM, Zheng YF, Peng B, Haapasalo M (2013). Current challenges and concepts of the thermomechanical treatment of nickel-titanium instruments. J Endod.

[12] Bryant ST, Thompson SA, al-Omari MA, Dummer PM (1998). Shaping ability of ProFile rotary nickel-titanium instruments with ISO sized tips in simulated root canals: part 2. Int Endod J.

[13] Ersev H, Yilmaz B, Ciftçioğlu E, Ozkarsli SF (2010). A comparison of the shaping effects of 5 nickel-titanium rotary instruments in simulated S-shaped canals. Oral Surg Oral Med Oral Pathol Oral Radiol Endod.

[14] Gergi R, Rjeily JA, Sader J, Naaman A (2010). Comparison of canal transportation and centering ability of twisted files, Pathfile-ProTaper system, and stainless steel hand K-files by using computed tomography. J Endod.

[15] Schäfer E, Tepel J, Hoppe W (1995). Properties of endodontic hand instruments used in rotary motion. Part 2. Instrumentation of curved canals. J Endod.

[16] Chirila M, Suciu I, Dimitriu B, Maru N, Ionescu E, Croitoru GF, Amza O (2020). Performance evaluation on Rotary Preparation of Root Canal by Beginner Operators. J Med Life.

[17] Burroughs JR, Bergeron BE, Roberts MD, Hagan JL, Himel VT (2012). Shaping ability of three nickel-titanium endodontic file systems in simulated S-shaped root canals. J Endod.

[18] Pacheco-Yanes J, Gazzaneo I, Pérez AR, Armada L, Neves MAS (2019). Transportation assessment in artificial curved canals after instrumentation with reciproc, Reciproc Blue, and XP-endo Shaper Systems. J Investig Clin Dent.

[19] Bergmans L, Van Cleynenbreugel J, Wevers M, Lambrechts P (2001). Mechanical root canal preparation with NiTi rotary instruments: rationale, performance and safety. Status report for the American Journal of Dentistry. Am J Dent.

[20] Nagaratna PJ, Shashikiran ND, Subbareddy VV (2006). In vitro comparison of NiTi rotary instruments and stainless steel hand instruments in root canal preparations of primary and permanent molar. J Indian Soc Pedod Prev Dent.

[21] Pinheiro SL, Araujo G, Bincelli I, Cunha R, Bueno C (2012). Evaluation of cleaning capacity and instrumentation time of manual, hybrid and rotary instrumentation techniques in primary molars. Int Endod J.

[22] Katge F, Patil D, Poojari M, Pimpale J, Shitoot A, Rusawat B (2014). Comparison of instrumentation time and cleaning efficacy of manual instrumentation, rotary systems and reciprocating systems in primary teeth: an in vitro study. J Indian Soc Pedod Prev Dent.

[23] Ramazani N, Mohammadi A, Amirabadi F, Ramazani M, Ehsani F (2016). In vitro investigation of the cleaning efficacy, shaping ability, preparation time and file deformation of continuous rotary, reciprocating rotary and manual instrumentations in primary molars. J Dent Res Dent Clin Dent Prospects.

[24] Celik D, Taşdemir T, Er K (2013). Comparative study of 6 rotary nickel-titanium systems and hand instrumentation for root canal preparation in severely curved root canals of extracted teeth. J Endod.

[25] Wu MK, Fan B, Wesselink PR (2000). Leakage along apical root fillings in curved root canals. Part I: effects of apical transportation on seal of root fillings. J Endod.

[26] Bertrand MF, Lupi-Pégurier L, Médioni E, Muller M, Bolla M (2001). Curved molar root canal preparations using Hero 642 rotary nickel-titanium instruments. Int Endod J.

[27] Shaikh SM, Goswami M (2018). Evaluation of the Effect of different Root Canal Preparation Techniques in primary Teeth using CBCT. J Clin Pediatr Dent.

[28] Hasheminia SM, Farhad A, Sheikhi M, Soltani P, Hendi SS, Ahmadi M (2018). Cone-beam computed Tomographic Analysis of Canal Transportation and Centering ability of single-file Systems. J Endod.

[29] Kumar BS, Pattanshetty S, Prasad M, Soni S, Pattanshetty KS, Prasad S (2013). An in-vitro evaluation of canal transportation and centering ability of two rotary Nickel Titanium systems (twisted files and Hyflex files) with conventional stainless Steel hand K-flexofiles by using spiral computed tomography. J Int Oral Health.

